# Balancing novelty and familiarity: a person-centered approach to openness to change and conservation values in tourism

**DOI:** 10.3389/fpsyg.2026.1860571

**Published:** 2026-10-01

**Authors:** Ingvild H. Blomstervik, Svein Ottar Olsen, Ana Alina Tudoran

**Affiliations:** 1 School of Business and Economics, UiT The Arctic University of Norway, Tromsø, Norway; 2 Department of Economics and Business Economics, Aarhus University, Aarhus, Denmark

**Keywords:** familiarity, latent profile analysis, novelty, personal values, person-centered approach

## Abstract

Understanding how personal values shape preference formation is a central concern in personality and social psychology, and increasingly relevant to tourism research. This study examines how openness to change (self-direction, stimulation, and hedonism) and conservation values (conformity, tradition, and security) are associated with preferences for novelty and familiarity in vacation experiences. Unlike prior variable-centered studies, this research adopts a person-centered approach to identify profiles based on within-person value structures. Using latent profile analysis on data from 498 US tourists, we identified four distinct value profiles: Tradition-focused, Stimulation-focused, Strong, and Weak. The Tradition-focused profile aligns with conservation values, favoring familiar experiences, while the Stimulation-focused profile, aligned with openness to change, favors novel experiences. The Strong profile places high importance on both openness to change and conservation values, showing the most balanced preference for new and familiar elements among the four profiles. The Weak profile places low importance on all values, reflecting flexible vacation preferences. These findings challenge the traditional dichotomy of novelty and familiarity, demonstrating that individuals can simultaneously hold preferences for both. By showing that openness to change and conservation values can be jointly important within individuals, this study extends the theoretical understanding of compatible and conflicting values and contributes to person-centered research on individual differences. Applied to tourism, these insights offer practical implications for tailoring vacation experiences to diverse value profiles.

## 1 Introduction

Tourism research has long recognized that tourism experiences arise from a blend of novelty and familiarity (Cohen, 1972), with recent studies demonstrating that the optimal level of interestingness occurs when both elements are balanced (Larsen et al., 2019). The literature has extensively explored the attraction of novelty, emphasizing its role in motivating travel and its potential to elicit unexpectedness, evoke positive emotions, and create memorable tourism experiences (Skavronskaya et al., 2020, 2021). In contrast, the role of familiarity in enhancing tourists' engagement in new experiences has received less attention (Clarke and Bowen, 2018) despite growing evidence of its importance in promoting long-term place attachment and loyalty (Clarke and Bowen, 2021). It is, therefore, relevant to explore the balance between the vacation preferences of novelty and familiarity. Are they conflicting or opposite ends of a continuum (Bello and Etzel, 1985), or can an individual simultaneously seek both, suggesting they are independent constructs with distinct antecedents and consequences (Larsen et al., 2019). Given the enduring relevance of both novelty- and familiarity-seeking behaviors in tourism (Stylidis and Terzidou, 2026), it is crucial to investigate the individual differences underlying these conflicting or balancing vacation preferences.

Research on novelty in tourism is dominated by concepts from personality psychology, such as sensation seeking (Zuckerman, 1979), stimuli seeking (Berlyne, 1960), or variety seeking (McAlister, 1982). These personality-based accounts explain why individuals seek novelty, but not why some individuals seek familiarity, or why some individuals might seek both. Understanding individual attitudes and behaviors is also fundamentally connected to personal values, an individual difference closely related to personality traits (Parks-Leduc et al., 2015). Although personal values are gaining growing attention in tourism (Maghrifani et al., 2024; Zhang et al., 2024), it has been only minimally explored in existing research on novelty (Blomstervik and Olsen, 2022), still not tested at the individual level (Blomstervik and Olsen, 2026). Schwartz's (2012) theory of personal values presents values in a circular structure, where values situated close to each other represent compatible values and those depicted further apart represent conflicting values. The value dimensions of openness to change (self-direction, stimulation, and hedonism) are relevant to the preferences for novelty as they encourage behaviors that seek new and exciting experiences. The value dimension of conservation (conformity, tradition, and security) is relevant to the preferences for familiarity as these values emphasize stability and the known.

Linked to behavior, compatible values may promote certain behaviors, whereas values inhibiting the same behavior are presented as conflicting (Skimina et al., 2019). Additionally, values only influence actions when they are relevant in the context and important to the individual (Schwartz, 2012, p. 4). For example, traveling on a novel wilderness safari can be motivated by stimulation values (openness to change) but may come at the expense of security values (conservation). In contrast, traveling to a familiar, established resort can be driven by security values (conservation) but can compromise the values of stimulation (openness to change). This example illustrates how the values of stimulation and security can represent conflicting values related to either novelty or familiarity vacation preferences.

However, circular structures with compatible and conflicting values show inconsistencies and vary across cultural regions (Steinmetz et al., 2012). Drawing on the previous example, the novel wilderness safari might be part of a group tour accompanied by an experienced guide, which can again align with security values. Simultaneously, a familiar established resort might include accommodation in igloos placed in an arctic environment, supporting the value of stimulation. In these examples, the values of stimulation and security are compatible and covered by vacation trips offering elements of both novelty and familiarity. This suggests that the strict conventional treatment of compatible and conflicting values may not always hold, opening the possibilities of individuals also driven by a balanced set of values, pursuing vacations that offer both novelty and familiarity (Borg et al., 2017).

Knowing which values are relevant to novelty and familiarity does not show how strongly these values might influence individual preferences. Recent research indicates that the importance individuals assign to their values profoundly affects their behavior, with stronger values leading to more frequent alignment with corresponding behaviors (Lee et al., 2022). In social psychology, importance is a key feature of attitude strength, together with certainty, ambivalence, and accessibility, impacting decisions and resistance to change (Howe and Krosnick, 2017; Petty et al., 2023). Attitude strength theory extends to values, suggesting that value strength is realized when a value is important, clear, and relevant to the individual (Howe and Krosnick, 2017; Schwartz, 2012). To further advance the understanding of the circular structure of values, this study explores how values with different levels of importance, ranging from high to low, can be structured within an individual and how these structures are associated with preferences for novelty and familiarity. In doing so, this study contributes to the existing literature on the relationship between important values and attitude strength (Boninger et al., 1995; Eaton and Visser, 2008), extending it through profiles with a subset of values relevant to novelty and familiarity.

Over the last decade, person-centered, within-person, and intra-individual approaches have attracted growing interest (Howard and Hoffman, 2018). Recently, several researchers have used this approach to study personal values (Borg et al., 2017; Gollan and Witte, 2014; Magun et al., 2015; Skimina et al., 2021). This approach allows for testing the circular structure of values within individuals and facilitates the investigation of groups with unique value structures in relation to other constructs (Magun et al., 2015; Howard and Hoffman, 2018). Although individuals within each group may share similarities, the groups are distinct, with different combinations of traits, states, or features. It is theoretically interesting to test whether individuals hold only compatible values, or whether some also deviate by holding conflicting values as well. Among the different person-centered analytic methods, cluster analysis and latent profile analysis stand out. Latent profile analysis is recognized for its flexibility, robustness, and interpretability (Magidson and Vermunt, 2002). This makes it a superior choice for our study, which delineates the profiles of individuals who ascribe similar levels of strength to specific values and examines how these alignments are associated with vacation preferences.

This study contributes to personality and social psychology by linking value content, value importance, and person-centered approach to preferences for novelty and familiarity. Regarding value content, rather than treating openness to change and conservation as strictly opposing poles within Schwartz's (2012) circular structure, this study demonstrates that these values can be jointly important within the same individual. This extends the theoretical understanding of compatible and conflicting values (Borg et al., 2017; Skimina et al., 2021). Regarding value importance, how strongly individuals hold openness-to-change and conservation values patterns their preferences for novelty and familiarity. These preferences function as independent dimensions, rather than opposite ends of a single continuum. Regarding the person-centered approach, this study demonstrates how individuals hold unique structures of values, in terms of both content and importance. More specifically, the present study identifies profiles of individuals with unique value structures in content and importance, based on openness to change (stimulation, self-direction, and hedonism) and conservation (tradition, conformity, and security), and examines how these profiles are associated with preferences for novelty and familiarity. Applied to tourism, this contributes to the existing literature on personal values and novelty (Maghrifani et al., 2024; Skavronskaya et al., 2021) by suggesting that novelty and familiarity preferences can coexist, and must be defined and measured as independent constructs.

## 2 Theory

### 2.1 Preference for novelty and familiarity

Early research on “novelty” in tourism literature uses the term to describe new, contrasting, and unfamiliar experiences (Bello and Etzel, 1985; Crompton, 1979; Pearson, 1970). Today, novelty is acknowledged as a critical factor in creating memorable tourism experiences by enhancing emotions, triggering memories, and providing tourists with unique and engaging experiences that leave a lasting impact (Skavronskaya et al., 2020). Specifically, the appraisal of novelty has the potential to elicit unexpectedness, which can eventually lead to surprise and other positive and negative emotions (Skavronskaya et al., 2021). Chark et al. (2020) found that novelty-seeking tourists with a higher need for novelty on their vacations also have a higher travel frequency than those with a lower need for novelty. Following this, others point to how novelty-seeking tourists are more likely to experience flow states associated with deep enjoyment and engagement in activities, which, in turn, enhance their intentions to engage in creative travel experiences (Kuo and Chang, 2024).

Familiarity with a tourism experience encompasses both previous experiences and information or knowledge of the experience (Baloglu, 2001). Recent studies have demonstrated how familiarity influences cognitive and affective destination image (Stylidis et al., 2020; Stylidis and Terzidou, 2026). Others point to how familiarity is associated with the intention to visit and recommend a destination (Casali et al., 2021), but this impact is only positive up to a certain degree before reaching saturation (Chi et al., 2020). Pearce (2012) introduced familiar tourists, who are depicted as tourists who prefer to visit both their homes and familiar places. These tourists have extensive knowledge of the setting and return to places where they have had emotional and personal connections in their lives. Building on this, Clarke and Bowen (2018, 2021) demonstrate how familiar tourists frequently vacate to familiar places, with patterns of visitation that are individually bonded. However, this does not exclude the possibility of familiar tourists choosing new destinations for their vacations. Furthermore, these tourists often extend their experiences to family members and advocate for a familiar place within their wider social network.

In tourism literature, elements of novelty and familiarity are evident in the tourism experience. Larsen et al. (2019) found that an optimal level of interestingness is achieved when both novel and familiar elements are present in the experience simultaneously for novelty-seekers and familiarity-seekers. This means that a tourist experience is more likely to be perceived as interesting when it offers a blend of new and exciting elements and familiar and comfortable aspects, regardless of whether the tourist prefers novel or familiar experiences. Others point to how novelty and familiarity capture different dimensions of the tourism experience (Guan et al., 2022) and influence tourists' behavior independently of each other (Toyama and Yamada, 2012). Recent literature also points to how novelty and familiarity influence destination image differently (Stylidis and Terzidou, 2026). This emphasizes why novelty and familiarity are the focal points of research. Figure 1 proposes a conceptual framework developed for this study that pictures novelty and familiarity as independent constructs, in which individuals can desire both to different degrees simultaneously without compromising each other. Therefore, it is important to test whether and how preferences for novelty and familiarity are associated with a combination of individual differences in personality, values, or other possible fundamental causes of these preferences (see Blomstervik and Olsen, 2022 for a recent update). Two of the preference alternatives are balanced, with either low familiarity and novelty preferences or high familiarity and novelty preferences. The remaining two are focused, with either high preferences for familiarity and low preferences for novelty or low preferences for familiarity and high preferences for novelty (see Figure 1).

**Figure 1:**
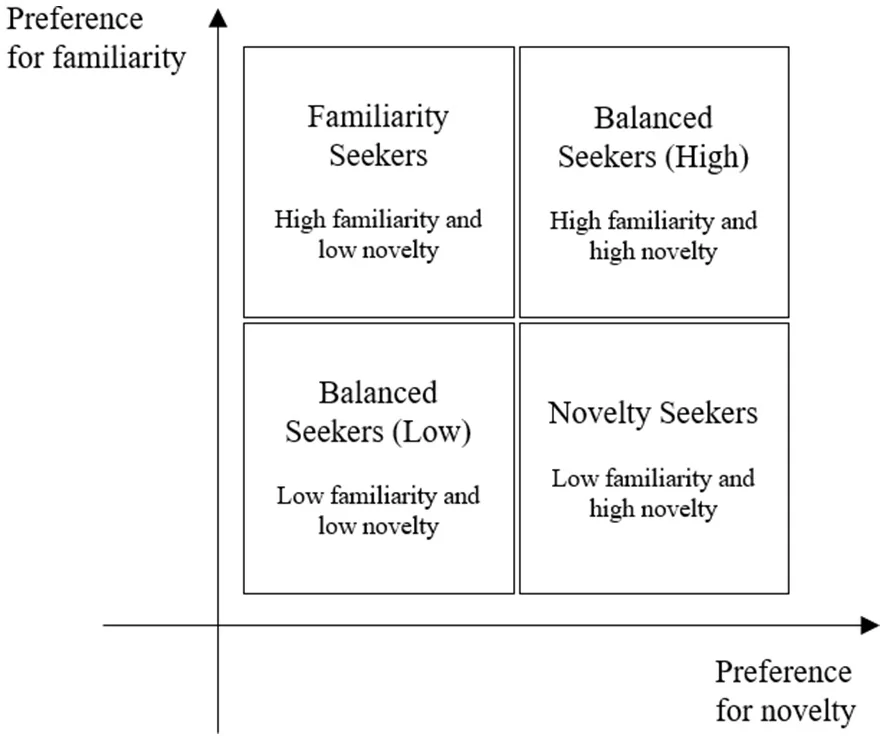
Possible combinations of preference for novelty and familiarity.

### 2.2 Personal values

Multiple theoretical approaches, models, and measurement typologies have been developed in various research domains to understand personal values (Sagiv and Schwartz, 2022). Presently, the main contemporary theories used when studying values comprise the functional theory of values, the psycho-lexical approach, and the theory of basic human values. The functional theory of values posits that values serve two main functions, namely individual needs and goals (Gouveia et al., 2014), while the psycho-lexical approach assumes that values are expressed in terms considered important in a language (De Raad et al., 2016). Finally, the theory of basic human values understands values as broad goals that drive actions and act as guiding principles in life (Sagiv and Schwartz, 2022, p. 518). This theory was selected for the present study because it structures values in relation to one another, a feature the functional theory of values and the psycho-lexical approach do not provide in the same form. It is also widely utilized across various fields of social science, including tourism (Kim, 2020).

The theory of basic human values emphasizes that values have distinct features, including their form, such as beliefs, desirability, stability, rationale, importance, and structure (Schwartz, 2012). For instance, values are beliefs closely tied to emotions. This means that when individuals hold certain values, they become filled with feelings when activated. Furthermore, values are described as inherently desirable, as they represent positive and worthy goals that can motivate behavior when trying to achieve them. Additionally, they are described as stable, meaning that they are consistent and enduring through specific and general behavior over time and across various contexts similarly to personality traits (Parks-Leduc et al., 2015). Personal values are also described as guiding principles, meaning that they can be used as reasoning or support for behavior, and serve as standards for the evaluation of objects. Finally, according to Schwartz (2012), they are structured by their relative importance to each other. The features of importance and structure will be discussed further.

#### 2.2.1 Values are organized by importance and strength

As noted above, personal values are organized by value importance, which refers to the varying significance or priority that individuals attribute to their personal values (Lee et al., 2022). Value importance is a key factor in understanding the relationship between personal values and behavior (Schwartz, 2012). Lee et al. (2022) propose that the strength of the relationship between personal values and behavior varies systematically across the distribution of value importance. Specifically, individuals attributing high importance to a specific value are associated with more frequent engagement in behaviors that align with this value, and this relationship strengthens as the level of value importance increases. Conversely, individuals who attribute low importance to certain values engage in behaviors that align with these values less frequently, and this relationship weakens as the level of value importance decreases. This makes value importance significant to the discussion as it links personal values and behavior in the tourism context.

Values are beliefs linked to affect and differ from general attitudes in their format of evaluation (Schwartz, 2012; Schwartz et al., 2012). While values are relatively stable and evaluated by their importance, general attitudes, which are more specific to time and context, are primarily evaluated by valence and extremity (Eagly and Chaiken, 1993). Despite this distinction, values are the basis for our evaluations because they underlie our attitudes (Schwartz, 2012, p. 16). Moreover, personal values and attitudes are theoretically connected by another defining feature, namely strength in their association with goals as a guiding principle in life or in association with the attitude object. Attitude strength refers to the extent to which an attitude influences thinking and behavior across situations (Howe and Krosnick, 2017, p. 328). Common attributes or features of attitude strength include importance, certainty, ambivalence, accessibility, and knowledge (Howe and Krosnick, 2017). These attributes can sometimes interact to form weaker attitudes, such as when one is certain of ambivalent attitudes (Petty et al., 2023).

The attribute of importance is particularly relevant as it reflects the subjective evaluation of the significance an individual ascribes to their attitudes (Boninger et al., 1995; Eaton and Visser, 2008). Importance can be measured similarly to personal values by asking individuals to indicate how important an attitude object is to them, and the importance ascribed to the attitude objects can also be compared (Howe and Krosnick, 2017). However, Luttrell and Sawicki (2020) point to how importance and other attitude attributes are predictors of attitude strength and should not be mistaken for attitude strength itself, emphasizing that attribute strength should be defined based on durability and impact. Durability refers to attitude resistance to change and stability over time, whereas impact is associated with influence on behavior and thinking (Krosnick and Petty, 2014). This distinction is central to the present study. The present study captures value importance, using “strong” and “weak” descriptively to refer to the relative importance individuals place on a value, not to its durability, certainty, or resistance to change.

#### 2.2.2 Compatible, conflicting or balancing structures of value importance

According to Schwartz (2012), personal values are presented within a circular structure in which the positioning of distinct values is determined by their compatibilities and conflicts. Values that align closely promote compatible motivations, potentially encouraging both specific and general behaviors. Conversely, values that are diametrically opposed to each other often reflect conflicting motivations in value importance and are likely to inhibit similar behaviors. This assumption of compatibility and conflict has been confirmed in several empirical studies (Skimina et al., 2021).

However, there is evidence of individuals holding conflicting values that are not necessarily believed to be congruent with each other, proposing a balanced set of values. Recent studies have explored the relationship between mountainous environments and personal values, which opens the possibilities of alternative value structures (Stieger et al., 2022). The results show that individuals living in challenging mountain terrain may experience pressure that forces them toward conflicting value orientations. Adapting to these environments requires openness to new experiences and changes. However, living under these conditions demands being dedicated to ensuring safety, maintaining stability, and upholding the current order. This implies that individuals settling in mountainous environments likely possess the characteristics of openness and security values. Similar preferences are found among individuals doing backcountry skiing (Marengo et al., 2017). Despite being characterized as sensation seekers and risk-takers performing activities that might compromise their safety, they still place emphasis on familiarizing themselves with the ski area and ensuring the availability of safety equipment. This behavior reflects the interplay between seeking thrill and adventure related to stimulation and ensuring personal safety and preparedness related to security. Research investigating work choices show evidence of conflicting value preferences occurring (Arieli et al., 2020). Individuals engaged in realistic work types commonly associated with practical and physical tasks involving machines, animals, and outdoor activities display this conflict. They often have a strong desire for security values but are also inclined toward activities that provide a sense of excitement related to stimulation values. This demonstrates that, even in work life, individuals may navigate between the need for secure and stable conditions while simultaneously seeking variety and challenges. This indicates how individuals with conflicting values may perceive these values as having a balanced structure. These examples are drawn from mountain living, backcountry skiing, and occupational settings, and it remains an open question whether the same pattern extends to tourism preferences.

Others again stress that individuals may attribute similar importance to conflicting values but that they typically do not pursue them simultaneously in specific situations (Skimina et al., 2021). Then again, in situations that are not chosen by oneself but controlled by others, the traditional circular value structure with compatible and competing values is difficult to identify (Skimina et al., 2018). In these situations, behavior is driven by external pressure and conflicting values can occur and conformity is often of relevance. Linked to vacation behavior, this can possibly arise when traveling with a group of people. This might also open the discussion about how individuals pursue a balanced set of personal values.

#### 2.2.3 Openness and conservation as relevant values for novelty and familiarity

For values to have impact on behavior they must be accessible, meaning that they need to be relevant to the situation and context to be activated (Skimina et al., 2019). In the context of vacation travel, values relevant to this discussion are those of self-direction, stimulation, and hedonism, which form the dimension of openness to change, together with the values of conformity, tradition, and security, which shape the dimension of conservation. These values are particularly relevant when considering preferences for novelty and familiarity because they represent conflicting motivational goals that directly influence travel behaviors (Skimina et al., 2019). While other value dimensions, such as self-enhancement, could influence travel preferences, they do not directly address the core tension between novelty and familiarity as explicitly as the two dimensions of openness to change and conservation. This focused selection means the two dimensions must be interpreted in relation to each other, chosen because they are proposed to be the values most relevant to novelty and familiarity.

Self-direction, stimulation, and hedonism values express openness to change, where independence, excitement, and pleasure are important goals (Schwartz, 2012). These values include aspects such as freedom, creativity, varied lives, daring, and enjoying life. They are often associated with behaviors linked to engagement in creative and exciting activities, providing self-development, and sensual gratification (Skimina et al., 2019). Such behaviors include writing, reading, learning, visiting new places, and dining at restaurants (Schwartz and Butenko, 2014; Schwartz et al., 2017). In tourism, research highlights how individuals holding these values can fulfill them by engaging in unique experiences that provide opportunities for involvement and learning (Ballantyne et al., 2021) and going on exciting holidays (Ye et al., 2017). Individuals who prioritize these values are also found to spend more financially on recreation activities compared to others (Lee et al., 2019). Building on this, it is reasonable to believe that these values are compatible with a preference for novelty when traveling, as they address the need for stimulation and excitement.

Conformity, tradition, and security express conservation and motivation for upholding existing norms and maintaining current conditions. This includes aspects of stability and harmony. These values are linked to behavior avoiding interpersonal conflict, and activities associated with personal health and security, such as physical and work activities (Skimina et al., 2019). Other concrete examples include avoiding conflicts and arguments, obeying rules and laws, respecting religion, and taking steps to avoid getting sick (Schwartz and Butenko, 2014; Schwartz et al., 2017). In tourism-related contexts, conformity, tradition, and security values exhibit a preference for meticulously organized vacations (Ye et al., 2017). Similarly, Watkins and Gnoth (2011) found that tourists who prioritize security tend to avoid unpredictable elements during their travels, instead seeking prearranged package tours accompanied by a guide that offers them the anticipated levels of security and convenience. This is further justified in a recent study showing how individuals valuing conservation values are more likely to exhibit motivations related to seeking assurance in their travel experiences, including travel with travel agencies and guided tours (Maghrifani et al., 2024). These values are believed to be compatible with a preference for familiarity, answering the need for stability and the known.

#### 2.2.4 Person-centered approach to personal values

Traditionally, the exploration of personal values has predominantly used inter-individual approaches, which also fall into the category of between-person or variable-centered approaches. Howard and Hoffman (2018) described these approaches by focusing on the interplay between a given set of variables, often using values as factors to analyze both correlations and regression weights across individuals in a sample. The benefit of this approach is that it provides a straightforward and generalizable understanding of the relationships between variables. However, downsides point to its limitations, including a lack of specificity and a limited understanding of subgroups and individual differences (Howard and Hoffman, 2018). Later studies shifted their focus, highlighting the need to study values using the intra-individual approach, also known as the within-person or person-centered approach (Borg et al., 2017; Gollan and Witte, 2014; Skimina et al., 2021). This approach provides detailed insights into subpopulations and captures individual differences, allowing for a more comprehensive analysis (Howard and Hoffman, 2018).

Applying this to research on personal values allows for the analysis of value structures at the individual level, capturing both value content and value importance. At the content level, this approach investigates whether individuals hold only the compatible values the circular structure predicts, or deviate from this by also holding conflicting values simultaneously. It also shows how the importance individuals ascribe to their values can be associated with individual preferences, behaviors, and decision-making. When extracting subgroups, different analytical methods can be used, but cluster analysis and latent profile analysis are preferred because of their flexibility, robustness, and interpretability (Magidson and Vermunt, 2002) and are used in this study. Other studies applying a similar approach ascribing individuals based on personal values in different subgroups have, for example, labeled profiles according to their strong/weak or personal/social value priorities when all value dimensions are included (Leite et al., 2021; Magun et al., 2015). Others have termed the profiles using the main dimensions of Schwartz's theory, such as openness to change, self-enhancement, conservation, and self-transcendence (Leite et al., 2021). Previous studies use the person-centered or intra-individual approach to cluster personal values based on their profiles on all value dimensions in Schwartz's theory. Our study differs from these studies because it profiles six openness and conservation values as the most relevant in association with novelty in tourism.

## 3 Materials and methods

### 3.1 Participants and procedure

Data collection was executed in December 2023, resulting in a representative sample of 498 US individuals. The Prolific platform (http://www.prolific.co) was used to collect respondents, who were compensated based on the time taken to finish the survey, with an average reward of £9.85 per hour. The representative sample was obtained through Prolific, ensuring a similar demographic distribution of age, sex, and ethnicity of the US population, based on data from the US Census Bureau. The university ethics committee approved data collection after reviewing and ensuring the ethical requirements of the study protocol.

Data quality was ensured through several procedures. Prolific assigns each participant a unique profile ID, which prevented duplicate participation. Respondents who completed the survey in an implausibly short time, or who showed disengaged response patterns, were excluded prior to analysis.

The respondents were between 18 and 70 years of age, consisting of 50% women, 48.6% men, and 1.4% reported others. In the sample, 65.1% had completed higher education, 64% had either part-time or full-time employment, and 24.1% had retired. Regarding travel companions, 17.9% preferred traveling alone, whereas the majority (44.2 %) traveled with their partner or spouse. Additionally, 11.0% traveled with friends, and 15.1% with other family members.

### 3.2 Measurements

The question of how exactly the human mind reacts to survey questions and produces an answer is complex, and question framing shapes the response given (Tourangeau et al., 2000). This is reflected in an extensive body of theoretical and methodological research on the measurement of personal values, spanning different scaling and framing approaches, each with distinct advantages and disadvantages (Ovadia, 2004).

*Values* were measured using the revised, validated Portrait Values Questionnaire (PVQ-RR) by Schwartz et al. (2012). The original scale used a third-person approach, asking the respondents to rate how similar the person is to them. To facilitate the respondents' understanding, the scale was adapted with a first-person approach e.g., “*For me, it is important..*.”. The first-person format asks respondents to rate their own values directly, rather than first judging how similar they are to a person described in the third person. This adaption has also been applied in other studies (Giménez and Tamajón, 2019). The complete structure has four dimensions, whereas our study uses openness to change and conservation dimensions, which are relevant to novelty. The values were measured based on self-direction (four items), stimulation (four items), hedonism (three items), conformity (four items), tradition (four items), and security (four items). Value items were answered applying rating scale with 9 response categories, where 1 = “strongly disagree” and 9 = “strongly agree” (see Table A1).

In this study, we focus on vacation preferences for novelty and familiarity. *Novelty* was measured from an attitude perspective (like, want, enjoy, and prefer) following the novelty-seeking scale by Lee and Crompton (1992) inspired by the dimension of “change from routine.” Four items were applied measuring *novelty*, namely “*I like to experience new things on vacation*,” “*I want to experience new things on vacation*,” “*I enjoy experiencing new things on vacation*,” and “*I prefer to explore something new on vacation*.” The three other dimensions—thrill, boredom alleviation, and surprise—were excluded, as they may measure other constructs not explicitly related to the core of novelty. *Familiarity* was measured using similarly adapted wording to compare concepts. The four items were: “*I like to experience familiar things on vacation*,” “*I want to experience familiar things on vacation*,” “*I enjoy experiencing familiar things on vacation*,” and “*I prefer to explore something familiar on vacation*.” Novelty and familiarity items were assessed using a 7-point Likert scale, where 1 = “strongly disagree” and 7 = “strongly agree.”

### 3.3 Data analysis

Because the value items had been adapted from their original format, the measurement structure was not assumed but examined in two *steps* using IBM SPSS Statistics (version 29). Principal component analysis (PCA) with varimax rotation was conducted first, as a preliminary examination of the dimensionality of the adapted items, establishing that they resolved into the expected number of distinct dimensions before a measurement model was specified. Confirmatory factor analysis (CFA) was then used to test the hypothesized structure, in which items loaded on their theoretically assigned value dimensions, and to assess the adequacy and discriminant validity of the measurement model (reported in Section 4.1).

Latent profile analysis (LPA) was used to identify profiles of tourists ascribing similar strengths and/or weaknesses to self-direction, stimulation, hedonism, tradition, conformity and security. The profiling indicators were the mean-centered latent dimension scores derived from the confirmatory model. LPA belongs to the family of finite mixture models; whereas latent class analysis is suited to categorical indicators, LPA extends the framework to continuous variables (Nylund et al., 2007). Unlike distance-based methods such as *k*-means or hierarchical clustering, LPA uses model-based clustering, applying maximum likelihood estimation to fit a probabilistic model of the distribution of the data, thereby accounting for within-group variability and avoiding assumptions about the shape of clusters (McLachlan and Peel, 2000). Models were estimated in Latent GOLD version 5.10.21260 (Vermunt and Magidson, 2021).

Solutions from one to six profiles were estimated. Model selection was guided by information criteria—the Bayesian Information Criterion (BIC; Schwarz, 1978), Akaike Information Criterion (AIC; Akaike, 1974), consistent AIC (CAIC; Bozdogan, 1987) and integrated completed likelihood BIC (ICL-BIC; Biernacki et al., 2000)—together with indicators of classification quality (entropy *R*^2^, classification error and average posterior probabilities of assignment) and the theoretical and practical interpretability of the resulting profiles.

Sample size requirements for mixture models such as LPA are not governed by a single fixed threshold; the ability to recover the correct number of profiles depends jointly on sample size, the degree of separation between profiles, the number and reliability of the indicators, and the relative sizes of the profiles (Tein et al., 2013; Wurpts and Geiser, 2014). Monte Carlo work on class enumeration indicates that the information criteria employed here perform well at sample sizes in the region of *N* = 500 (Nylund et al., 2007), and applied guidance for LPA converges on samples of this magnitude as adequate for models with a small number of well-measured, theoretically specified indicators (Nylund-Gibson and Choi, 2018; Spurk et al., 2020). The present sample of *N* = 498, with six indicators, therefore falls within the range in which profile enumeration can be conducted with confidence.

To guard against reliance on a single methodological approach, the profile structure was additionally examined using hierarchical clustering. After the number of profiles was determined, a series of *t*-tests was conducted to examine differences among the profiles in vacation preferences for novelty and familiarity.

## 4 Results

### 4.1 Reliability and validity

Because several instruments were adapted for the present study (Section 3.2), the measurement structure was examined before any substantive analyses were conducted. PCA with varimax rotation was used first, as a preliminary examination of the dimensionality of the adapted value items; the extracted components accounted for 77.2% of the variance and reproduced the expected pattern of associations among the measured variables.

The measurement structure was then tested using CFA. The model showed acceptable fit (χ^2^(406) = 1293.793, χ^2^/df = 3.187, CFI = 0.922, TLI = 0.910, RMSEA = 0.066, SRMR = 0.052), and all items loaded significantly (*p* < 0.001) on their intended constructs, with loadings ranging from 0.626 to 0.932, above the recommended threshold of 0.50 (Hair et al., 2010).

Reliability and convergent validity were satisfactory for all eight constructs. Composite reliability ranged from 0.808 to 0.949 and Cronbach's alpha from 0.767 to 0.948, both exceeding the conventional threshold of 0.70, while average variance extracted ranged from 0.547 to 0.823, exceeding the 0.50 threshold (Table A1). Discriminant validity was supported by heterotrait-monotrait (HTMT) ratios of correlations, all of which fell below the 0.850 threshold, with a maximum of 0.676 between stimulation and hedonism (Table A2; Henseler et al., 2015). Of particular relevance to the present study, the HTMT ratio between novelty and familiarity was 0.236, indicating that the two constructs were empirically distinct rather than opposite poles of a single dimension, consistent with their treatment as independent constructs.

These results indicate that the adapted instruments performed satisfactorily in the present sample. The latent value dimension scores derived from the confirmatory model were carried forward to the latent profile analysis.

### 4.2 Latent profile analysis

#### 4.2.1 Determining the number of profiles

Drawing on the theoretical background, we aim to identify stable value profiles of individuals and their preferences for novelty and familiarity in tourism experiences. The profiling process utilized mean-centered latent value dimension scores extracted from the CFA (as explained above). Solutions from one to six profiles were estimated, with the one-profile model providing a baseline against which the presence of latent heterogeneity could be assessed. Model selection was guided by information criteria (BIC, CAIC, AIC, AIC3 and ICL-BIC), by indicators of classification quality (entropy *R*^2^, classification error and average posterior probabilities of assignment), and by the theoretical and practical interpretability of the resulting profiles. Fit statistics for all solutions are reported in Table 1.

**Table 1:** Goodness of fit for one- to six-profile solutions.

Profile	LL	Npar	BIC	CAIC	AIC	AIC3	ICL-BIC	Max. BVR	Class. error	Entropy *R*^2^	Class sizes (%)
1-Profile	−5,173.055	12	10,420.636	10,432.636	10,370.109	10,382.109	10,420.636	230.735	–	–	100
2-Profiles	−4,752.522	45	9,784.521	9,829.521	9,595.044	9,640.044	9,925.718	176.960	0.059	0.795	52.7/47.3
3-Profiles	−4,565.393	78	9,615.213	9,693.213	9,286.787	9,364.787	9,814.202	73.370	0.081	0.811	46.7/29.1/24.2
4-Profiles	−4,393.353	111	9,476.082	9,587.082	9,008.706	9,119.706	9,679.980	40.566	0.078	0.844	42.2/20.7/20.2/16.9
5-Profiles	−4,284.410	144	9,463.146	9,607.146	8,856.819	9,000.819	9,710.467	28.653	0.098	0.841	29.3/23.1/21.0/13.6/13.0
6-Profiles	−4,182.971	177	9,465.217	9,642.217	8,719.941	8,896.941	9,696.950	26.441	0.086	0.867	23.7/21.2/16.4/15.2/12.4/11.1

LL, log-likelihood; Npar, number of parameters; BIC, Bayesian Information Criterion; CAIC, Consistent AIC; AIC, Akaike Information Criterion; ICL-BIC, Integrated Completed Likelihood BIC, which augments BIC with a penalty for classification uncertainty (Biernacki et al., 2000); BVR, bivariate residual. Lower values indicate better fit for all information criteria. Classification error and entropy *R*^2^ are not defined for the one-profile solution.

The information criteria did not converge on a single solution. The substantial improvement from the one-profile model (BIC = 10420.64) through the four-profile model (BIC = 9476.08) confirms pronounced latent heterogeneity in value structures. Beyond four profiles, however, the criteria diverged. AIC and AIC3 decreased monotonically across the range tested, a pattern commonly observed in mixture models given their comparatively light penalties for model complexity. BIC was lowest for the five-profile solution (9463.15), although the difference from the four-profile solution (12.94 points) was small relative to the 33 additional parameters required. In contrast, both CAIC (9587.08) and ICL-BIC (9679.98) were minimized at the four-profile solution. ICL-BIC is particularly relevant in the present context, as it augments BIC with a penalty for classification uncertainty and is therefore appropriate when the objective is the recovery of well-separated, substantively interpretable groups rather than density estimation alone (Biernacki et al., 2000).

Indicators of classification quality pointed in the same direction. The four-profile model showed the lowest classification error of all solutions tested (0.078, compared with 0.098 for the five-profile solution) and an entropy *R*^2^ of 0.844, with average posterior probabilities of assignment ranging from 0.915 to 0.937, all well above the conventional threshold of 0.80.

To address the limitations inherent in any single methodological approach, we supplemented our analysis with hierarchical clustering, a distance-based method that provides an alternative perspective on data segmentation (Hennig et al., 2015). This technique does not require a predetermined number of clusters, providing a complementary check on the structure suggested by LPA. The dendrogram from the hierarchical clustering (see Figure A1) visually illustrates the aggregation of individual observations into distinct groups. By observing the substantial height at which mergers occur, we identify four natural groupings.

The four-profiles identified by this solution (subsequently labeled Tradition-focused, Stimulation-focused, Strong values and Weak values, and described in detail in Section 4.2.2), provided the reference against which the alternative solutions were evaluated. In the *five-profile solution*, three of the four original profiles were reproduced with essentially the same value configurations: the Stimulation-focused profile (21.0%; openness to change above the sample mean, conservation well below it), the Strong values profile (13.6%) and the Weak values profile (13.0%), the latter two comprising fewer cases but showing the same patterning. The Tradition-focused profile, however, was not reproduced in the same form. In its place the solution contained a sharper conservation-oriented profile (23.1%; conformity = 1.28, tradition = 1.64, security = 0.71, with openness values close to the sample mean) and a larger profile (29.3%) whose means lay close to the sample average on all six dimensions (range −0.54 to −0.06). This latter profile (the largest in the five-profile solution) did not represent a distinct configuration of value priorities, but an undifferentiated position close to the sample average on every dimension.

The *six-profile solution* showed the same tendency. The Stimulation-focused, Strong values and Weak values profiles were again recovered, with the Strong values profile comprising progressively fewer cases (11.1%). A profile with means close to the sample average on all six dimensions was again present and again among the largest (23.7%; range −0.69 to 0.11), while the conservation-oriented region of the solution was divided into two profiles distinguished principally by their degree of openness to change (stimulation = 0.76 and −1.00, respectively) rather than by qualitatively different value configurations. Beyond four profiles, therefore, additional profiles produced graded variants of the same underlying structure, together with a large group lacking a distinctive value signature.

The *four-profile solution* was retained on the combined basis of classification quality, the absence of significant improvement in fit beyond four profiles, the convergence of CAIC and ICL-BIC on this solution, and the alignment of the resulting profiles with the theoretical framework of Schwartz's personal values (Section 4.2.2). We note that the statistical criteria do not uniquely identify a single optimal solution, and that the four-profile model was therefore selected as the most defensible rather than the statistically dominant one.

#### 4.2.2 Description of profiles

Figure 2 lists the sizes and characteristics of the four profiles. In interpreting the profile means, positively centered means reflect a high priority assigned to the corresponding values, negatively centered means indicate low priority, and means close to zero indicate a priority close to the sample average. The four profiles show substantial heterogeneity in the values that individuals prioritize, which is essential for addressing our research objectives and consistent with the theoretical framework.

- The Tradition-focused profile is characterized by moderately positive scores on conservative values such as conformity (0.510), tradition (0.719), and security (0.271). At the same time, they are moderately negative toward self-direction (−0.219), stimulation (−0.264), and hedonism (−0.185). This pattern illustrates a moderate preference for conservation and tradition over openness to change values.
- The Stimulation-focused profile shows the lowest affinity for traditionalist (−2.076) and conformist values (−1.487) among all profiles, with significant negative scores for tradition (−2.076) and conformity (−1.487) and a minor negative score for security (−0.733). Conversely, they had positive attitudes toward stimulation (0.903), self-direction (0.506), and hedonism (0.468), indicating a strong inclination toward openness and experiential values.
- The Strong profile scored highest on tradition (1.878), conformity (1.314), and security (0.965), compared with the other three profiles. Simultaneously, this profile showed high scores for hedonism (1.087), stimulation (1.165), and self-direction (0.563). This pattern suggests a strong orientation toward values associated with conservation and stability while still being open to new experiences.
- The Weak profile exhibits low scores across all values, particularly on stimulation (−1.842), self-direction (−0.746) and hedonism (−1.427), compared with the other three profiles. This pattern may point to a tourist profile that is more neutral or casual toward the values of openness and conservation.

**Figure 2:**
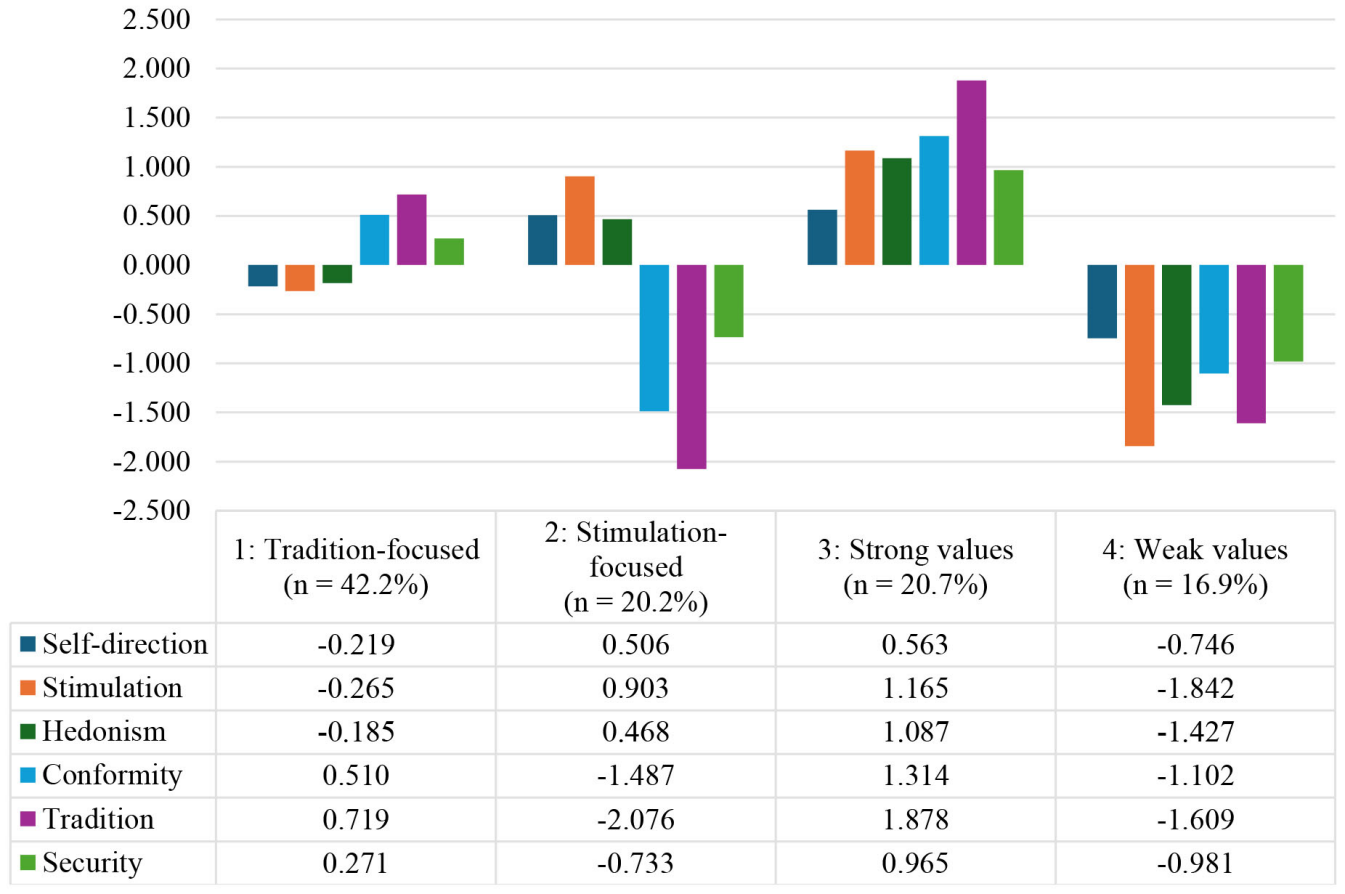
Comparison of the centered means (*M*) of values among the four profiles including pairwise comparisons and significance level. Significance level of differences between groups (*p*-values): Self-direction: 1 vs. 2 = <0.001; 1 vs. 3 = <0.001; 1 vs. 4 = <0.01; 2 vs. 3 = 0.55; 2 vs. 4 = <0.001; 3 vs. 4 = <0.001. Stimulation: 1 vs. 2 = <0.001; 1 vs. 3 = <0.001; 1 vs. 4 = <0.01; 2 vs. 3 = 0.02; 2 vs. 4 = <0.001; 3 vs. 4 = <0.001. Hedonism: 1 vs. 2 = <0.001; 1 vs. 3 = <0.001; 1 vs. 4 = <0.01; 2 vs. 3 <0.001; 2 vs. 4 = <0.001; 3 vs. 4 = <0.001. Conformity: 1 vs. 2 = <0.001; 1 vs. 3 = <0.001; 1 vs. 4 = <0.01; 2 vs. 3 = 0.55; 2 vs. 4 = <0.001; 3 vs. 4 = 0.14. Tradition: 1 vs. 2 = <0.001; 1 vs. 3 = <0.001; 1 vs. 4 = <0.01; 2 vs. 3 = 0.55; 2 vs. 4 = <0.001; 3 vs. 4 = 0.16. Security: 1 vs. 2 = <0.001; 1 vs. 3 = <0.001; 1 vs. 4 = <0.01; 2 vs. 3 = 0.55; 2 vs. 4 = <0.001; 3 vs. 4 = 0.34.

The statistical Wald tests indicate that each indicator contributes significantly to the model. Stimulation appears to be the most influential factor in differentiating between the profiles, followed by Tradition, Hedonism, and Conformity, based on the *R*^2^ values presented in Table 2. Security and Self-direction discriminate less, implying that these values are less definitive in clustering profiles, suggesting a more universal or homogeneous importance across groups. This homogeneity may reflect baseline agreement or consistency among all profiles regarding the fundamental need for security and autonomy, regardless of other value orientations. Thus, while specific values such as Stimulation and Tradition markedly influence profile distinctions, Security and Self-direction serve as common ground for stabilizing the value landscape across different individual structures.

**Table 2:** Wald test, *p*-value and *R*^2^ for the different values.

Values	Wald	*p*-value	*R* ^2^
Self-direction	162.2149	6.1e-35	0.2580
Stimulation	505.0452	3.8e-109	0.6093
Hedonism	398.6404	4.4e-86	0.4594
Conformity	258.3096	1.0e-55	0.4313
Tradition	356.4198	6.1e-77	0.4778
Security	234.9370	1.2e-50	0.3083

### 4.3 Describing value profiles in association with preferences for novelty and familiarity

This section explores the association between each profile and the preferences for novelty and familiarity with tourism experiences. *T*-tests were used to examine the differences between these preferences within each profile, and the significant results are presented in Table 3. The negative mean differences across all profiles indicated a high preference for novelty over familiarity. This pattern suggests that regardless of their value orientation, tourists in each profile are more inclined to seek new experiences than familiar ones. Figure 3 illustrates the profile preferences for novelty and familiarity and their associations, building on the association proposed in Figure 1.

- The Tradition-focused value profile exhibits the highest preference for familiarity (*M* = 4.713) compared to the other three profiles, and it is also higher than the overall average (*M* = 4.475). Additionally, they display an average novelty preference (*M* = 5.893), which is slightly lower than their overall preference (*M* = 6.094). This suggests that individuals in the Tradition-focused profile are more conservative and moderately open to new experiences yet still prefer familiar settings. This profile is closest to the high familiarity-low novelty preferences in Figure 1 compared to the other three profiles and represents a weakly focused value structure.
- The Stimulation-focused value profile shows a high novelty preference score (*M* = 6.593) and the lowest score for familiarity (*M* = 3.873) compared to the other three profiles. This indicates a clear preference for novelty over familiarity, with the largest mean difference of −2.719, underscoring the desire for new and less common travel experiences. This profile is closer to the high novelty and low familiarity preferences in Figure 1 than the other three profiles, representing a strong focused value structure.
- The Strong-value profile demonstrated the highest preference for novelty (*M* = 6.611) among the profiles, significantly exceeding the overall mean. Their familiarity preference scores (*M* = 4.609) are similar to the overall average. This indicates that individuals in this profile seek new and novel experiences yet still appreciate some elements of familiarity. This profile is closest to the high novelty and familiarity preferences in Figure 1 among the four profiles, showing the most balanced preference for novelty and familiarity, and represents a balanced and strong value structure.
- Finally, the Weak value profile illustrates more moderate preferences, with the lowest score for novelty (*M* = 5.342) and a low score for familiarity (*M* = 4.407) compared to the overall scores. The mean differences between preferences for novelty and familiarity within this profile were also the smallest among the four profiles (−0.935), indicating that individuals have more indifferent vacation preferences than the other profiles. This profile is closest to the low-novelty and low-familiarity preferences in Figure 1 compared to the other three profiles and represents a balanced and weak value structure.

**Table 3:** Profiles based on preferences for novelty and familiarity profiles including pairwise comparisons and significance level.

Profiles	Novelty (NO)	Familiarity (FA)	Mean differences (FA-NO)	*t*-value	df	*p*-value	95% confidence interval
Profile 1: Tradition-focused	5.893	4.713	−1.179	−11.039	212	< 0.001	(−1.390, −0.368)
Profile 2: Stimulation-focused	6.593	3.873	−2.719	−16.967	98	< 0.001	(−3.037, −2.401)
Profile 3: Strong values	6.611	4.609	−2.002	−12.412	104	< 0.001	(−2.322, −1.682)
Profile 4: Weak values	5.342	4.407	−0.935	−4.454	80	< 0.001	(−1.352, −0.517)
*Wald test*	67.585	21.813					
*p-value*	< 0.001	< 0.001					

Significance level of differences between groups (*p*-values): Novelty: 1 vs. 2 = < 0.001; 1 vs. 3 = < 0.001; 1 vs. 4 = < 0.01; 2 vs. 3 = 0.11; 2 vs. 4 = < 0.001; 3 vs. 4. = < 0.001. Familiarity: 1 vs. 2 = 0.36; 1 vs. 3 = < 0.001; 1 vs. 4 = < 0.01; 2 vs. 3 = < 0.001; 2 vs. 4 = < 0.001; 3 vs. 4 = 0.790.

**Figure 3:**
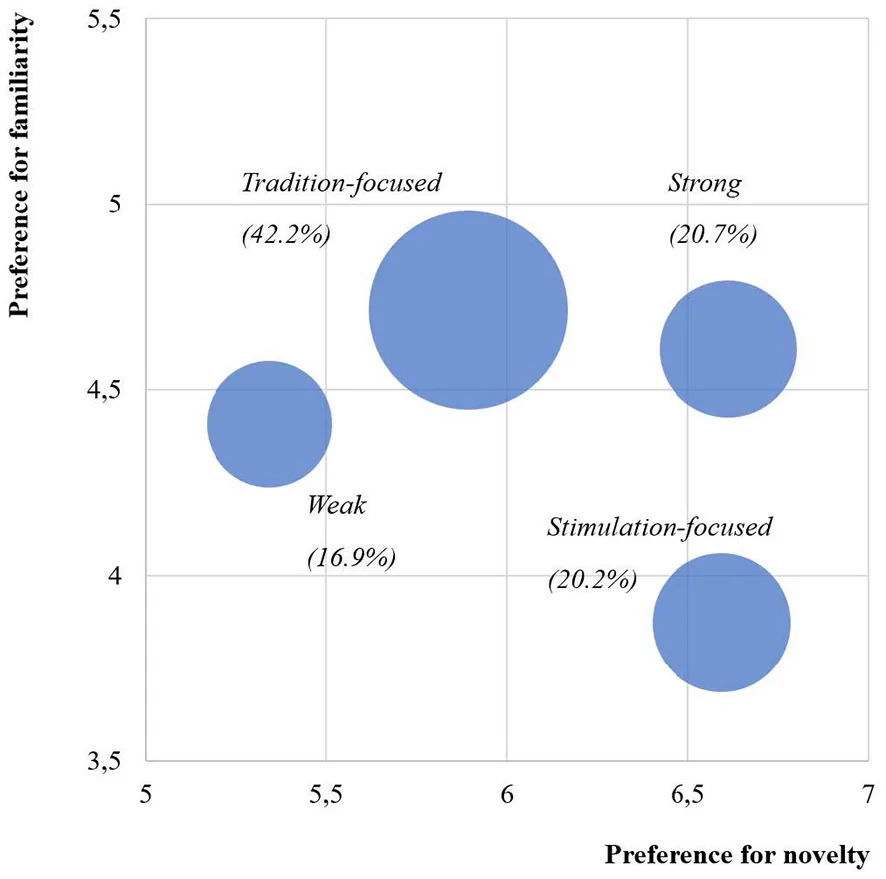
Comparison of the vacation preferences for the profiles found.

Figure 3 builds on the proposed assumptions regarding novelty and familiarity preferences presented in Figure 1. The positions of these profiles illustrate how they differ from each other. Tradition-focused and Stimulation-focused profiles are situated on opposite sides of the matrix, highlighting their contrasting preferences for either novelty or familiarity when compared to the overall mean. Similarly, the Strong- and Weak-value profiles demonstrated the most balanced preferences for novelty and familiarity, though at differing intensity levels (strong and weak).

### 4.4 Describing profiles based on sociodemographic characteristics

The differences between the profiles can be explained by their distinct sociodemographic characteristics presented in Table 4. Wald's test for the significance of covariates reveals that Age and Work are statistically significant. The Tradition-focused profiles and Strong values profiles exhibit a higher proportion of older participants especially in the age group above 60 (0.390 / 0.596), either retired or with other occupational tasks. In contrast, the Stimulation-focused and the Weak Values profiles show a higher proportion of younger participants with diverse occupational situations. The Weak-value profile is the profile with the highest proportion of full-time and part-time employed (1.309 / 2.025).

**Table 4:** Wald test of significance differences between profiles based on age and work.

Demographics	Profile 1: Tradition-focused	Profile 2: Stimulation-focused	Profile 3: Strong values	Profile 4: Weak values
Age				
18–25	−0.035	0.234	−0.452	0.253
26–35	−0.571	0.402	−0.031	0.200
36–45	0.011	0.517	−0.257	−0.271
46–59	0.205	−0.516	0.144	0.167
Above 60	0.390	−0.637	0.596	−0.349
*Wald test*	26.745			
*p-value*	0.009			
Work				
Full-time employee	−0.203	−0.825	−0,281	1.309
Part-time employee	−0.819	−0.683	−0.522	2.025
Student	−0.855	0.526	−0.439	0.768
Retired/unemployed	−0.321	−0.751	0.210	0.862
Other	2.198	1.733	1.033	−4.964
*Wald test*	21.09			
*p–value*	0.049			

Significance level of differences between groups (*p*-values): Age: 1 vs. 2 = 0.49; 1 vs. 3 = 0.002; 1 vs. 4 = 0.14; 2 vs. 3 = 0.008; 2 vs. 4 = 0.31; 3 vs. 4 = 0.28. Work: 1 vs. 2 = 0.43; 1 vs. 3 = 0.21; 1 vs. 4 = 0.021; 2 vs. 3 = 0.25; 2 vs. 4 = 0.058; 3 vs. 4 = 0.28.

## 5 Discussion and conclusions

This study advances our understanding of the interplay between personal values (Schwartz, 2012) and vacation preferences for novelty and familiarity in the tourism context (Larsen et al., 2019). By employing latent profile analysis, this research identifies four different individual profiles, namely the Tradition-focused (42.2%), Stimulation-focused (20.2%), Strong (20.7%), and Weak values (16.9%). Each profile demonstrates unique personal value structures, illustrating how values can be both compatible and conflicting (Skimina et al., 2019) but also focused and balanced (Marengo et al., 2017; Stieger et al., 2022) using a person-centered approach (Howard and Hoffman, 2018). These findings also offer an alternative understanding of the traditional perspective of novelty and familiarity as opposites, opening up discussions on the combinations of preferences for novelty and familiarity (Larsen et al., 2019; Toyama and Yamada, 2012).

When comparing the values overall, stimulation appears to be the most influential among the openness to change values in differentiating between profiles. Among the conservation values, tradition is most important in describing the profiles. This finding aligns with earlier studies (Schwartz et al., 2017; Skimina et al., 2019, 2021), which demonstrates that stimulation and tradition are conflicting values and might not be pursued to the same extent by the same individuals. Self-direction and security are the least influential values in distinguishing between profiles. This may be because these values are perceived as universal across all value profiles and serve as common ground.

The Tradition-focused profile strongly emphasizes conservation values (conformity, tradition, and security) while placing less importance on openness to change values (self-direction, stimuli, and hedonism). The Stimulation-focused profile contrasts this by attributing strong importance to openness to change values alongside lower importance to conservation values. These profiles align closely with Schwartz's (2012) conceptualization of compatible and conflicting values, where openness to change and conservation values are often presented as opposite value structures (Skimina et al., 2021). These profiles illustrate a focused value structure to varying degrees, desiring either stimulation or tradition.

The Strong profile distinguishes itself by placing high importance on both openness to change and conservation values. This profile challenges the traditional view that treats openness and conservation as conflicting value dimensions, illustrating that individuals consider both sets of values as important. This finding contributes to the discussion of individuals who maintain a balanced value set and are open to new experiences while simultaneously valuing conformity (Marengo et al., 2017; Stieger et al., 2022). In comparison, a Weak profile is characterized by a general undervaluation of all values, especially stimulation, hedonism, and tradition. This suggests a less defined or indifferent approach to their personal values, still also illustrating a weak balance between openness to change and conservation values. This pattern may reflect the complexity of assigning importance to values in situations not chosen by the individuals themselves, as contexts influenced by external factors may lead to less clear value priorities (Skimina et al., 2018). These results contribute to the existing literature suggesting the theoretical and empirical relationship between value relevance and attitude strength and importance (Boninger et al., 1995; Eaton and Visser, 2008).

The results of this study support the notion that treating preferences for novelty and familiarity as contrasting may be overly simplistic (Guan et al., 2022; Larsen et al., 2019). Consistent with this recent literature, our findings suggest that tourists do not exclusively prefer novel or familiar experiences but rather seek a blend that aligns with their personal values. Notably, the preference for novelty was higher than familiarity across all profiles, indicating a general inclination toward seeking new experiences. However, none of the profiles completely avoided familiarity, and the preference for familiarity was not uniformly low across all profiles. This suggests that all profiles exhibited some degree of preference for familiarity.

The Tradition-focused profile has a stronger preference for familiarity than the other profiles but individuals within this profile still also seek novelty. This aligns with the characteristics of familiarity-seeking tourists, who often travel to known destinations and are still interested in elements of novelty (Clarke and Bowen, 2018, 2021). The Stimulation-focused profile, exhibiting strong preferences for openness alongside lower scores for conservation values, represents the novelty-seeking tourist described in early literature (Crompton, 1979; Skavronskaya et al., 2020). This profile's focused preference for new experiences aligns with the notion that novelty can enhance emotions, trigger memories, and create memorable tourism experiences by adding elements that lead to unexpectedness and surprise (Skavronskaya et al., 2021). However, it is important to note that this profile does not oppose familiarity.

The Strong profile, characterized by high scores in both openness to change and conservation values, has the most balanced preferences of the four profiles (Larsen et al., 2019). This relative balance indicates a desire for vacations that combine new and exciting elements with familiar and comfortable aspects, more so than in the other profiles. Nevertheless, it aligns with the literature suggesting that individuals valuing conservation prefer familiar experiences, but also new experiences aligned with openness (Ballantyne et al., 2021; Maghrifani et al., 2024; Ye et al., 2017). In contrast, the Weak value profile, with a weak balanced structure with low scores across all values, may indicate a less defined or indifferent approach to vacation preferences. This suggests that some individuals do not strongly prioritize the values associated with either novelty or familiarity, potentially seeking vacation experiences that are convenient or available rather than those that align with their personal values. Overall, the results indicate that individuals with focused value structures on either openness to change or conservation tended to focus on vacation preferences for either novelty or familiarity. Individuals with more balanced value structures combining openness to change and conservation instead exhibited relatively more balanced vacation preferences combining novelty and familiarity.

The sociodemographic analysis adds depth to our understanding of these profiles, revealing that the Stimulation-focused profile shows a higher proportion of younger students. This demographic trend may reflect broader societal shifts toward valuing unique and authentic experiences, especially among younger tourists who prioritize excitement and adventure (Kuo and Chang, 2024). The Strong profile's higher proportion of older participants suggests that as individuals age, they may seek a relatively more balanced set of vacation preferences, valuing both new experiences and the comfort of the familiar.

In conclusion, this study makes significant theoretical contributions by extending Schwartz's theory of personal values to tourism preferences, offering an understanding of how individual value structures are associated with vacation preferences for novelty and familiarity. By identifying and analyzing the Tradition-focused, Stimulation-focused, Strong- and Weak-value profiles, this research provides insight into the relationship between openness to change and conservation values. Thus, it contributes to theory by illustrating that value profiles can be focused on either openness to change or conservation values, and that value profiles can also be balanced by combining openness to change and conservation values. This research additionally contributes to the theory by suggesting that tourists' preferences are not strictly motivated by either novelty or familiarity but reflect a spectrum where novelty and familiarity can be balanced to varying degrees, and aligned with the underlying personal values. Overall, this research advances the theoretical understanding of personal values in tourism, setting a foundation for future studies to delve deeper into the relationship between personal values and tourism experiences.

## 6 Practical implications, limitations and future research

The findings of this study highlight the importance of considering individual differences and personal values in shaping the preferences for novelty and familiarity in tourism experiences. This has practical implications for tourism marketing and management, which can benefit from recognizing and catering to the diverse value profiles and their balancing preferences for novelty and familiarity, offering more personalized tourist experiences. The practical suggestions below follow from the findings, they are illustrative examples, not empirically tested interventions, and should be evaluated by practitioners accordingly. For the tradition-focused profile, tourism providers should develop offerings that emphasize comfort, tradition, and security. Familiar destinations enriched with novel elements can engage this group, such as heritage tours incorporating new culinary adventures or cultural workshops. Marketing strategies should craft campaigns that resonate with their preference for tradition while introducing minor innovations, using, for example, storytelling to connect past and present experiences. The stimulation-focused profile requires experiences that prioritize adventure, novelty, and uniqueness. Providers should focus on promoting new and exciting destinations that offer unexpectedness and thrilling elements, such as activities triggering adrenaline or expeditions to unexplored natural wonders. Marketing strategies should focus on presenting these experiences using vibrant images and engaging narratives that communicate the thrill and excitement of discovering new and unexpected destinations. For the strong profiles, travel packages should balance novelty with familiarity, catering to their desire for both new experiences and familiar comforts. Examples include luxury accommodation paired with adventurous excursions. Marketing messages should emphasize the harmony between exploration and comfort, using testimonials and actual cases to illustrate how these balanced experiences can satisfy diverse preferences. The weak profile benefits from flexible and convenient travel options that do not heavily emphasize either novelty or familiarity. This approach appeals to those who prioritize practicality and convenience over specific value-driven experiences. Marketing strategies should highlight the ease and accessibility of travel options, focusing on practical benefits such as convenience and flexibility using straightforward messaging that appeals to their approach to travel.

Although this study provides valuable insights, we acknowledge its limitations, including its cross-sectional design and the self-reported nature of personal values and vacation preferences. Because the data are cross-sectional and self-reported, the associations reported here should not be interpreted as causal effects of personal values on vacation preferences. Since the sample is drawn exclusively from the United States, the findings should be interpreted as specific to this cultural context, and may not generalize directly to countries where value priorities or tourism norms might differ (Steinmetz et al., 2012). The profiles in this study reflect within-person priorities among openness-to-change and conservation values, rather than an individual's position within the full Schwartz value circle. Future research could extend this analysis to include the whole personal value circle proposed by Schwartz (2012). The link between personal values and personality traits has also been investigated by others and could provide additional insights into the analysis of preferences for novelty and familiarity. Moreover, individuals' vacation preferences are not limited to novelty and familiarity, and the list could be extended to include both uniqueness and authenticity. One limitation of this study is the potential for sampling bias due to the use of an online platform for data collection. The participants are compensated for their participation, which may influence their response behavior, providing answers to ensure completion and compensation. Future research could address this limitation by employing alternative sampling methods or platforms to validate the findings across different participant pools. Methodologically, while LPA offers valuable insights into data structures, it is inherently limited by its dependence on model specification, such as the number of profiles tested and the selection of statistical criteria (Marsh et al., 2009). The LPA assumption of conditional independence within profiles can also lead to overfitting and potential misinterpretation. To bolster our findings, we supplemented our LPA with additional tests, including distance-based clustering methods to verify the natural grouping of data. However, these methods also carry their own set of subjective methodological challenges (Hennig et al., 2015).

Future research could employ different research designs, measurement approaches or scaling procedures to explore how individuals' preferences for novelty and familiarity evolve, to gain deeper insights into the motivations behind these preferences, and to explore the structure of personal values. Additionally, exploring the role of emerging trends such as sustainable and responsible tourism in shaping these preferences could offer further contributions to the field. Investigating how different cultural context influences the association between personal values and vacation preferences could also enhance our understanding of tourist behavior.

## Data Availability

The raw data supporting the conclusions of this article will be made available by the authors, without undue reservation.
