# Supplementary material for: Balancing novelty and familiarity: a person-centered approach to openness to change and conservation values in tourism
Source: Front Psychol. 2026 Oct 1;17:1860571. doi: 10.3389/fpsyg.2026.1860571 (PMC13669110; doi:10.3389/fpsyg.2026.1860571)
Supplement: Supplementary file 1 [file Supplementary_file_1.docx]

*Table A1 Factor reliability and validity*

| Constructs and indicators | Loadings | CR | AVE | Cronbach’s Alpha |
| --- | --- | --- | --- | --- |
| *Self-direction* |  | 0.834 | 0.560 | 0.832 |
| … to form my views independently. | 0.626 |  |  |  |
| …to develop my own opinions. | 0.750 |  |  |  |
| …to make my own decisions about my life. | 0.837 |  |  |  |
| …to be free to choose what I do. | 0.764 |  |  |  |
| *Stimulation* |  | 0.844 | 0.576 | 0.840 |
| …to always look for different things to do. | 0.796 |  |  |  |
| …to do things that make life exciting. | 0.796 |  |  |  |
| …to have all sorts of adventures. | 0.762 |  |  |  |
| …to do things that add variation in my life. | 0.674 |  |  |  |
| *Hedonism* |  | 0.808 | 0.586 | 0.767 |
| …to have a good time. | 0.853 |  |  |  |
| …to enjoy the pleasures of life. | 0.776 |  |  |  |
| …to seize any opportunity to have fun. | 0.654 |  |  |  |
| *Security* |  | 0.870 | 0.627 | 0.864 |
| …that my country has stability. | 0.828 |  |  |  |
| …that my country protects itself from any threat. | 0.847 |  |  |  |
| …to be secure. | 0.770 |  |  |  |
| …to be safe. | 0.716 |  |  |  |
| *Tradition* |  | 0.924 | 0.752 | 0.923 |
| …to uphold traditional values. | 0.890 |  |  |  |
| …to uphold traditional ways of thinking. | 0.887 |  |  |  |
| …to follow family customs. | 0.842 |  |  |  |
| …to respect the traditional practices of my **culture**. | 0.850 |  |  |  |
| *Conformity* |  | 0.827 | 0.547 | 0.833 |
| …to abide by rules. | 0.658 |  |  |  |
| …to obey all laws. | 0.834 |  |  |  |
| …to avoid upsetting others. | 0.658 |  |  |  |
| …to never make other people angry. | 0.656 |  |  |  |
| *Novelty* |  | 0.949 | 0.823 | 0.948 |
| I like to experience new things on vacation. | 0.894 |  |  |  |
| I want to experience new things on vacation. | 0.932 |  |  |  |
| I enjoy experiencing new things on vacation. | 0.885 |  |  |  |
| I prefer to explore something new on vacation. | 0.918 |  |  |  |
| *Familiarity* |  | 0.948 | 0.820 | 0.947 |
| I like to experience familiar things on vacation. | 0.923 |  |  |  |
| I want to experience familiar things on vacation. | 0.931 |  |  |  |
| I enjoy experiencing familiar things on vacation. | 0.905 |  |  |  |
| I prefer to explore something familiar on vacation. | 0.860 |  |  |  |

*Notes:* CR = composite reliability; AVE = average variance extracted; model fit: x^2^ = 1293.793; *df* = 406.000; *x^2^/df* = 3.187; *CFI* = 0.922; *SRMR* = 0.052; *RMSEA* = 0.066 All the loadings are significant at the *** *p* < 0.001 level.

*Table A2 Heterotrait–monotrait (HTMT) ratio of correlations*

|  | Novelty | Familiarity | Self-Direction | Stimulation | Hedonism | Conformity | Tradition |
| --- | --- | --- | --- | --- | --- | --- | --- |
| *Novelty* |  |  |  |  |  |  |  |
| *Familiarity* | 0.236 |  |  |  |  |  |  |
| *Self-Direction* | 0.329 | 0.039 |  |  |  |  |  |
| *Stimulation* | 0.582 | 0.094 | 0.405 |  |  |  |  |
| *Hedonism* | 0.417 | 0.033 | 0.472 | 0.676 |  |  |  |
| *Conformity* | 0.039 | 0.193 | 0.090 | 0.152 | 0.247 |  |  |
| *Tradition* | 0.019 | 0.272 | 0.008 | 0.207 | 0.224 | 0.574 |  |
| *Security* | 0.158 | 0.135 | 0.359 | 0.230 | 0.392 | 0.581 | 0.476 |

*Figure A1 Hierarchical clustering dendrogram*
